# Green Synthesis of Zeolitic Imidazolate Frameworks: A Review of Their Characterization and Industrial and Medical Applications

**DOI:** 10.3390/ma15020447

**Published:** 2022-01-07

**Authors:** Mahboobeh Shahsavari, Peyman Mohammadzadeh Jahani, Iran Sheikhshoaie, Somayeh Tajik, Abbas Aghaei Afshar, Mohammad Bagher Askari, Parisa Salarizadeh, Antonio Di Bartolomeo, Hadi Beitollahi

**Affiliations:** 1 Department of Chemistry, Faculty of Science, Shahid Bahonar University of Kerman, Kerman 7616913439, Iran; mahboob92sh@gmail.com (M.S.); shoaie@uk.ac.ir (I.S.); 2 School of Medicine, Bam University of Medical Sciences, Bam 7661771967, Iran; mjpeyman@yahoo.com; 3 Research Center of Tropical and Infectious Diseases, Kerman University of Medical Sciences, Kerman 7616913555, Iran; s.tajik@kmu.ac.ir (S.T.); abassaghaeiafshar@gmail.com (A.A.A.); 4 Department of Physics, Faculty of Science, University of Guilan, Rasht 4199613776, Iran; mbaskari@phd.guilan.ac.ir; 5 High-Temperature Fuel Cell Research Department, Vali-e-Asr University of Rafsanjan, Rafsanjan 7718897111, Iran; p.salarizadeh@vru.ac.ir; 6 Department of Physics “E. R. Caianiello” and “Interdepartmental Center NANOMATES”, University of Salerno, 84084 Fisciano, SA, Italy; 7 Environment Department, Institute of Science and High Technology and Environmental Sciences, Graduate University of Advanced Technology, Kerman 7631885356, Iran

**Keywords:** green synthesis, zeolitic imidazolate framework (ZIF), sensor, metal–organic frameworks (MOFs)

## Abstract

Metal organic frameworks (MOF) are a class of hybrid networks of supramolecular solid materials comprising a large number of inorganic and organic linkers, all bound to metal ions in a well-organized fashion. Zeolitic imidazolate frameworks (ZIFs) are a sub-group of MOFs with imidazole as an organic linker to metals; it is rich in carbon, nitrogen, and transition metals. ZIFs combine the classical zeolite characteristics of thermal and chemical stability with pore-size tunability and the rich topological diversity of MOFs. Due to the energy crisis and the existence of organic solvents that lead to environmental hazards, considerable research efforts have been devoted to devising clean and sustainable synthesis routes for ZIFs to reduce the environmental impact of their preparation. Green chemistry is the key to sustainable development, as it will lead to new solutions to existing problems. Moreover, it will present opportunities for new processes and products and, at its heart, is scientific and technological innovation. The green chemistry approach seeks to redesign the materials that make up the basis of our society and our economy, including the materials that generate, store, and transport our energy, in ways that are benign for humans and the environment and that possess intrinsic sustainability. This study covers the principles of green chemistry as used in designing strategies for synthesizing greener, less toxic ZIFs the consume less energy to produce. First, the necessity of green methods in today’s society, their replacement of the usual non-green methods and their benefits are discussed; then, various methods for the green synthesis of ZIF compounds, such as hydrothermally, ionothermally, and by the electrospray technique, are considered. These methods use the least harmful and toxic substances, especially concerning organic solvents, and are also more economical. When a compound is synthesized by a green method, a question arises as to whether these compounds can replace the same compounds as synthesized by non-green methods. For example, is the thermal stability of these compounds (which is one of the most important features of ZIFs) preserved? Therefore, after studying the methods of identifying these compounds, in the last part, there is an in-depth discussion on the various applications of these green-synthesized compounds.

## 1. Introduction

According to the literature, zeotypes have been considered the primary technological, nano-porous, inorganic substances, with more than 180 framework topologies. In addition to aluminosilicate zeolites, researchers have identified several zeolites, such as inorganic materials, in the 1980s and 90s, including transition metal phosphates (MeAPOs) and aluminium phosphates (AlPOs) [1,2,3]. Moreover, experts in the field have performed numerous studies on the development new classes of zeolitic structures on the basis of hybrid metal organic framework materials (MOFs). This research has been done toward enlarging the size of pores, improving their functions, and discovering new catalytic and sorption features [4].

Over the last years, researchers have much considered metal organic porous materials due to their attractive uses in separation, molecular recognition, and catalysis [5]. Nonetheless, they have encountered challenges in constructing open MOFs, which include the enhancement and modulation of pore dimensions for creating porous metalorganic materials of higher stability. Even though researchers have proposed multiple strategies for increasing the size of pores via the enlargement of organic moieties, these methods result in frameworks’ interpenetrative instability [6]. In addition, MOFs offer benefits, such as superior structural architectures, larger surface areas, stronger host-to-guest interactions, and potent uses in drug delivery, catalysis, separation and gas sorption, and storage. Notably, the methodology for creating a rigid, stable MOF with larger pores is based on the expansion of zeolitic topologies via producing organic, metal tetrahedral building-blocks. Therefore, researchers have selected four coordinate transition metals for the tetrahedral atoms (T) of zeolites and imidazolates as the linkers or vertices (X) of T. They hope that the resultant metal organic polymer may include an open framework with the zeolite or zeolite-like topologies. However, using imidazolate as a linker cannot be randomly chosen and must be deliberately selected because of the orientation of both the coordinating nitrogen atoms of imidazolate, with reference to each other at an angle of 1448°, similar to the 1458° angle of oxygen coordination in zeolites [7]. A concern has been raised regarding the creation of porous zeolite-like metal organic frameworks (ZMOFs) [8], wherein transition metals replace the tetrahedrally coordinated Si or Al atoms and the organic ligand links, replacing the oxygen bridges in zeolites. Researchers have also provided several MOFs with zeolite-analogous frameworks. Compared with the common, neutral MOF substances, ZIFs enjoy anionic frameworks and many of them have been considered to be charge-compensated by the extra framework ions in their cavities, which may be ion exchanged by alkali metals [8]. Current studies have shown the synthesis of nearly 20 ZIFs, such that one of their subsets, in fact, has a similar framework topology with zeolites; numerous ZIFs consist of Co or Zn ions when combined with functionalized imidazolate anions or imidazolate [4]. ZIFs have very good chemical and thermal stability and do not have Lewis and Brønsted acid sites, which can catalyse olefins’ polymerization within their pores [9].

There are diverse procedures for synthesizing ZIFs. Researchers have provided ZIF-based materials through hydrothermal/solvothermal synthesis practices in organic solvents [10]. These materials include the solvothermal reactions of a solution consisting of ligands and metal salts in organic solvents (for example: ethanol and N,N-dimethylformamide (DMF)) or mixtures of organic solvents and water. With respect to the above processes, expenses are increased by the use of conventional organic solvents such as DMF, resulting in risks for safety and the environment [11].

Simple decomposition of several organic solvents is observed, which results in the generation of numerous waste by-products that impact the features or functionalities of the intended products. Even though it is possible to recycle multiple organic solvents and reuse them in a number of circumstances [12], experts in economic and environmental areas believe that the commercial and industrial feasibility of MOFs uses [13] is hindered by toxic and detrimental solvents [14]. Hence, an attractive means for the utilization of various MOFs would be to devise a green path for the proper fabrication of yolk-shell structures and multi-functional ZIF [15]. Moreover, numerous researchers have addressed the development of other techniques for mitigating and eliminating the application and production of detrimental organic solvents in MOF synthesis [16,17], which include the solvent-free method [18], aerosol route [19], micro-wave irradiation as well as ultra-sound assisted procedures [20,21]. The above methods forgo the utilization of the detrimental solvents in the MOFs’ synthesis; and, they enjoy very good stability, adjustable porosity, higher rate of production, and continual generation in the resulting MOFs [22,23]. Nonetheless, they suffer from lower generality and require delicate synthetic methods and instruments [24,25].

Our review examines ZIFs synthesized by green techniques. The IUPAC has defined the concept “green chemistry”, which may be briefly described as inventing, designing, and applying chemical procedures and products for reducing or eliminating the application and production of detrimental materials [26].

In order to have a sustainable production of MOFs, researchers must consider one of the green and affordable production processes, a number of them are the utilization of inexpensive, recyclable, and renewable starting metal salts and acid linkers, less solvent or green solvents such as water, affordable energy resources, and proper low-risk synthetic techniques like lower pressure and temperature [27].

## 2. Synthesis and Characterization Methods

### 2.1. Green Methods for Synthesizing ZIFs

It is possible to adapt the twelve principles of green chemistry for synthesizing MOFs [28]. The mentioned criteria deal with the basic procedural dimensions, which include the application of the safer reagents, nontoxic solvents, renewable feedstock, energy efficiency, and waste removal, as well as bio-degradable final products. In addition, it is possible to evaluate the green and scalable synthesis of MOFs with regard to biocompatible building blocks, lower energy inputs, safer reaction media (like super-critical solvents and water), continual strategies of production, and performance design of MOFs via theoretical anticipations [20,29]. It is notable that ZIFs have been considered one of the novel classes of MOFs with organic linkers and metal ions [30].

Researchers have conducted numerous studies on developing a green and cost-effective synthesis process for producing ZIFs to decrease the utilization of organic solvents and the environmental effects thereof [31].

Investigators have also designed diverse preparation techniques, such as the green solvents [32], organic solvent-based routines [33] as well as solvent free [34] routines for generating ZIF-based substances. Now, the green synthesis of ZIFs is presented.

#### 2.1.1. Solvent Evaporation Synthesis Method

Solvent evaporation has been considered as a robust and strongly directional area. For example, Kim and Libera revealed the possible use of the rate of solvent evaporation from the solution cast thin films of block copolymers for manipulating the growth and orientation of copolymer assemblies [35]. Furthermore, researchers have provided steam assisted conversion (SAC) and the solvent evaporated conversion (SEC) for preparing characteristic MOFs and ZIFs with a specific solvent recovery method through zeolite. Out of the green synthesis methods, researchers have identified SAC’s potential efficiency benefits [36,37], such as lower waste disposal, lower reactor size, and less use of a template, as well as continual production for synthesizing numerous MOFs and zeolite.

Based on the research, solvents contribute importantly to several synthesis methods but many of them are too costly and detrimental to the environment [38,39]. Thus, it is necessary to present an effective solvent recovery by a cost-effective green synthesis method.

In their study, Chen et al. proposed one of the efficient solvent recovery approaches on zeolite for preparing ZIF-8 using SAC and SEC. They showed zeolite action as a medium for loading and transporting solvent that stops the solvents’ condensation. Moreover, SEC is capable of separating the process into two phases of SAC and solvothermal synthesis, affording a multistage reaction system for obtaining various products at all stages. Diffusion of organic ligands to metal sites in the steam environment is observed in the SAC process, which causes a fast reaction rate due to higher concentrations of the reactant. It has been found that the steam recovery method of the zeolite may efficiently recycle the solvent and offers a green procedure wherein the intended dry products may be directly applied. Moreover, researchers examined polar molecule NH_3_ adsorption of ZIF-8 at moist and dry conditions and observed a stable structure of ZIF-8 in the moist and pure NH_3_ conditions. Therefore, the same absorption features of NH_3_ and H_2_O of ZIF-8 could be compared with other MOFs that have stable structures and high water uptake with a potential in the NH_3_ adsorption uses [40].

#### 2.1.2. Ionothermal Synthesis Method

Ionothermal synthesis for preparing porous materials is one of the novel methods [41,42]. As a result of the absence of vapor pressure from the ionic liquid (IL), it is possible to perform ionothermal synthesis in open vessels under normal pressure, which prevents the risks of high autogenous pressures in a sealed autoclave. It should be mentioned that ILs are used as solvents and thus provide template cations around which the organic frameworks are requested [43]. Another study has proposed microwave heating technology as one of the new types of green chemical methods with extensive uses in numerous chemical synthesis areas [44,45].

Yang et al. used a new technique combining microwave energies (microwave-assisted ionothermal synthesis) and ionothermal synthesis for synthesizing ZIF-8. The method is aimed at the fast production of the adsorption substances, a combination of the benefits of IL and microwave heating, which is followed by acceptable results for high-energy efficiency and safe production, as well as a lack of pollution of the environment [46].

#### 2.1.3. Hydrothermal Synthesis Method

This method represents the synthesis via chemical reactions in an aqueous solution that is above the water boiling point. In fact, researchers hope that hydrothermal synthesis of the coordination frameworks result in a shift towards commercial production at high-production rates by decreasing the associated production expenses and environmental impacts [47].

The cheapest, most sustainable, and safest metal salts with which to synthesize MOFs have been proposed to be metal hydroxides, acetate, and oxides that offer environmentally benign anions. Moreover, researchers have synthesized Zn-based ZIF-8 in water via a hydrothermal path according to a green synthesis strategy that removes detrimental solvents and does not involve the application of surfactants. Misran et al. performed synthesis at room temperature that has been accompanied by a simplified hydrothermal procedure with palm oil-derived fatty alcohols (PODFA), which has 12 carbon chain in different solvents. It has been found that their mechanism is the same as that of the water–alcohol insoluble monolayer interfaces at the liquid-to-gas interaction. Therefore, the miscibility of the Zn^2+^ ions and deprotonated methylimidazole linkers are elevated by increasing the solvents’ polarity, which results in greater interaction with the metal ions and, thus, the creation of smaller unit cells [48].

Bombyx mori silk essentially contains silk sericin and silk fibroin (SF) [49]. Multiple researchers have addressed the development of SF-derived composites, adapted for biomedical and biotechnological utilizations. In addition, several studies demonstrated the structural merits of MOFs, such as larger specific surface areas, adaptable surface chemistry, and adjustable pore size, which are very valuable in functionalizing SF. Furthermore, MOFs@SF have exhibited greater potential for application in the bio-associated areas due to the respective dual-biocompatibility [50,51,52].

In their study, Song et al. addressed the development of a ZIF-8@SF linear composite using a simplified, green, and sustainable method. Upon ZIF-8 feeding, researchers did not find any considerable consequences for silkworms. The as-prepared composite had a maximum strain of 28% with 3% ZIF-8, which is 17% greater than that of natural SF. Finally, the results have shown the attractiveness of the above in-vivo feeding method to modify SF for commercial production [53].

According to a study in the field, sodalite, with cubic Im3m crystal symmetry, is six-membered (6R) with a pore size of 0.28 nm. In addition, the greatest diameter of the void in the framework is 0.63 nm and, thus, is a candidate for separating water and hydrogen [54]. Furthermore, ZIF-8 enjoys a sodalite zeolite-type topology, wherein the ZnN_4_ tetrahedral via imidazolate linkers form big pores of 11.6 Å that are linked by small apertures of 3.4 Å [55].

In their recent study, Zeng et al. indicate a ZIF-8@sodalite composite with the cor@shell structure that has been synthesized by the fast in-situ growth synthesis method. Hence, sodalite zeolites have been initially synthesized from the alkali-activated kaolin and ZIF-8 NPs have been on the exterior surface of the sodalite zeolites from 2-Methylimidazole (Mim) and zinc nitrate hexahydrate in methanol. Their study has been the first to investigate the in-situ synthesis of cor@shell ZIF-8@sodalite composites with hierarchical microporous and mesoporous structures at room temperature [56].

#### 2.1.4. Rapid Synthesis of Hierarchical Porous ZIFs

In this stage, ZIFs from the organic amine-based system have been synthesized. In this regard, Duan et al.’s study presents a green versatile method, through using organic amines as supra-molecular templates (organic amine template) for the fast synthesis of hierarchical porous ZIFs (ZIF-61, ZIF-90, and ZIF-8) at room pressure and temperature. In fact, organic amines play 2 roles in the synthesis process; (1) a protonation agent for deprotonating the organic ligands that simplify ZIF crystals’ formation, and (2) a structure directing agent for directing the formation of mesopore and or macropore. According to the analyses, the shortest synthesis period in this method has been one minute at room temperature and pressure while the common solvothermal procedures involve a synthesis time of 24 h at the high pressure and temperature, which reveals the prominent contribution of the organic amines to the accelerated creation of ZIF-8 that fulfils the affordable and environmental-friendly needs to synthesize ZIF [57,58].

Moreover, Zhang et al.’s study dealt with the synthesis of 2 hierarchical porous ZIF-8 and ZIF-90 at room temperature and pressure over 60 s with the use of N, N-diethylethanolamine as the template. They have found the high space time yield (STY) to be 1.59 × 104 kg m^−3^ d^−1^, which is greater than in other studies. The report actually offers a modern method for easily synthesizing hierarchical porous ZIFs with higher STY [59].

#### 2.1.5. Supercritical CO_2_ Synthesis Method

By implementating numerous green chemistry principles, super-critical carbon dioxide (scCO_2_) has appeared as an attractive reaction media for the preparation of chemical compounds and substances [60,61].

In addition to environmental merits, the physical and chemical features of scCO_2_ may offer multiple synthetic benefits, including: (1) it is possible to tune the liquid-like density via simple changes in pressure or temperature, which influence reaction equilibrium and solubility; (2) its gas-like viscosity and diffusivity may make the chemical reaction kinetics faster by enhancing the mass and heat transfer; and (3) its near-zero surface tension may simplify the surface wetting and penetration of the porous matrices that influence chemical reaction paths and interfacial energies. Moreover, it is possible to avoid common separation procedures via the rapid quenching of a synthesis by depressurization, which probably decreases agglomeration, creates more acceptable nano-structures, and enhances the surface areas [62].

Furthermore, Sinnwell et al.’s investigations address ZIF-8 synthesis in scCO_2_. They have shown that in-situ powder X-ray diffraction (XRD), ex-situ microscopy, and simulation offer a detailed vision of the formation of ZIF-8, as well as intermediary ZnO@ZIF-8 composites in non-traditional solvents. Therefore, researchers capitalized on the in-situ XRD capacities and revealed correlations of the rate of ZIF-8 formation with the surface area of the templating ZnO source. Then, they analysed the kinetics thereof and showed the effect of small changes to isobaric temperature on the variables crucial in the reaction pathway [63].

Consequently, Marrett et al.’s study offered a scalable and quick path to synthesizing ZIF-8 from a metal oxide via the use of scCO_2_ as a reaction medium, which eliminates the requirement for catalytic additives or aqueous or organic solvents. One of the unexpected results has been the quantitative conversion of ZnO to grams of distinctive ZIFs in minutes, with 100 g of common ZIF-8 achievable in 1 h [64].

#### 2.1.6. Electrospraying Synthesis Technique

Electrospraying has been proposed as one of the popular electrochemical techniques such that electrostatic charging produces fine liquid droplets. In fact, researchers have employed electrospraying for producing droplets with positive charges in a low, dielectric organic solvent medium, which collide and merge electrostatically [65].

Another study, conducted by Konno et al., employed a novel method to synthesize ZIF-67 crystals. According to the liquid electrospraying procedure, it is possible to improve mono-dispersity and morphology of ZIF-67 via electrospraying in a liquid phase that creates fine-liquid droplets as a reaction field. Their technique has been introduced as one of the forms of the environmental-friendly size controlled synthesis without any requirement for an organic synthetic solution, high concentration, temperature control and additives like surfactants [66].

#### 2.1.7. Rapid and Simplified Method

Diverse ZIF-8 synthetic techniques like hydro/solvothermal, sonochemical, electrochemical and microwave assisted paths for fabricating ZIF-8 at the temperatures in ranges between room temperature up to 200 °C in various solutions have been presented [67].

In their study, Cravillon et al. applied a simplified procedure for producing ZIF-8 nanocrystals. They mixed Mim and Zn(NO_3_)_2_ in methanol at room temperature [68]. It should be noted that these researchers took ideas from earlier investigations and provided various new synthesis techniques using additives such as trimethylamine [69], sodium formate [70], n-butylamine [71], and polyamine [72] in an organic-based solution system for optimizing preparation conditions and controlling the size and morphology of ZIF-8. Nonetheless, organic solvents have been considered to be costly and poisonous and, finally, research has seen the removal of several organic solvents, such as DMF, from pores of ZIF-8 crystals [39]. Notably, Pan et al.’s report indicated substantial synthesis of ZIF-8 in water at room temperature without the use of conventional additives [73].

**Figure 1:**
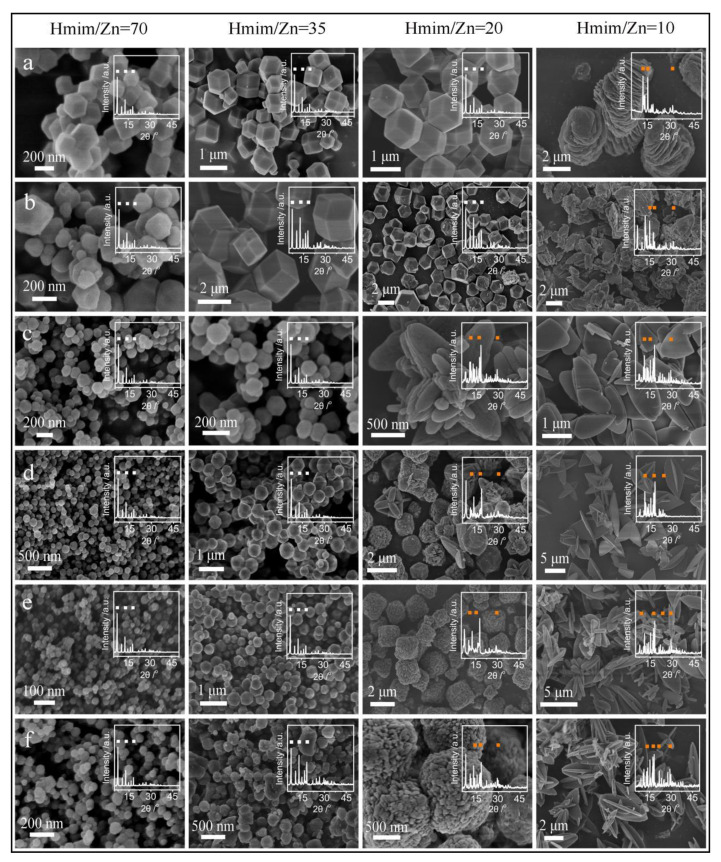
PXRD (powder X-ray diffraction) patterns and SEM (scanning electron microscopy) images (inserted) of the products provided from various ratios of Mim/Zn, beginning with (**a**) Zn(OAc)_2_, (**b**) ZnSO_4_, (**c**) Zn(NO_3_)_2_, (**d**) ZnCl_2_, (**e**) ZnBr_2_, and (**f**) ZnI_2_ [74].

Moreover, Jian et al.’s report demonstrated the substantial synthesis of ZIF-8 in water with the use of six zinc sources, under various conditions and at room temperature, without the use of conventional additives. During the synthesis process, researchers observed the easy control of the size of particles and shape of the resultant ZIF-8 materials using Mim content. According to their analyses, longer synthesis time promoted the detailed crystallization of ZIF-8 (Figure 1) [74].

Lo et al. have been the first to address the production of 110-nm ZIF-90 particles using a water–alcohol-based system and the impact of viscosity with an optimal H_2_O/tert-butanol/glycerol/PVP system. Their proposed green synthesis has been reported to be quick and reliable in comparison with common organic solvent-based procedures. Moreover, this water/polymer/alcohol system resulted in lower sizes of ZIF-90 particles, of nearly 100 nm, such that the particles could be employed in biomedical and industrial utilizations as a result of lower cytotoxicity and a less-toxic synthetic method [75].

The synthesis of ZIF-7 microcrystals in the exposure of aqueous media to a concentrated ammonia atmosphere has been performed by Ebrahimi and Mansournia at room temperature in a short time. They also examined stability and demonstrated the considerable chemical and thermal durability of the products. Finally, these researchers have shown the higher effectiveness of their method as compared with other synthetic techniques [76].

In addition, Wu et al. deal with the synthesis of a bio-mimetic mineralization path for synthesizing enzyme–MOF composites with horseradish peroxidase (HRP) and cytochrome c (Cyt c), as well as Candida Antarctica lipase B (CALB) as their model enzymes. Their method has been proposed as a green chemistry method of preparing immobilized enzymes based on MOFs for achieving higher enzyme stability under harsh conditions. When exposed to protein-denaturing solvents, such as dimethyl formamide, dimethyl sulfoxide, ethanol, and methanol, the enzyme/ZIF-8 composites exhibit 100% of their activity. Indeed, the free enzymes maintain 20% of their original activity under similar conditions. Their investigation shows the very high protective impact of ZIF-8 shells on enhanced enzyme stability under very harsh conditions [77].

While conventional synthesis methods are widely used, the fields of mechanical, sonochemical, and electrochemical synthesis, as well as microwave synthesis, are emerging. Researchers have demonstrated these methods to be applicable for some compounds, often under milder reaction conditions, yielding materials with different particle sizes and properties. This could be of interest for the up-scaling of syntheses and the application of ZIFs. However, the predominantly known compounds have been reproduced by these alternative pathways, and the reproducibility of these synthesis methods must be established. Great care must be taken with the actual energy input by various artificial methods. One method that is probably overlooked due to the complexity of the equipment and chemicals involved is ZIF electrochemical synthesis. Especially when working at high reaction temperatures, this method can lead to new ZIF structures with unusual metals in unusual oxidation states. A wide range of distinct preparation approaches, such as ionic liquids, molecular precursors, or in-situ bond synthesis has been performed in the last two decades. Finally, we want to raise a concern about comparisons of results reported on the synthesis of ZIFs. Great care must be exerted not only in synthesis but also in its activation and characterization in order to allow for reliable comparisons.

### 2.2. Characterization

In general, Fourier transform infrared spectroscopy (FT-IR), powder X-ray diffraction (PXRD) structure identification, N_2_ adsorption–desorption isotherms (textural features of the pore volume or surface area), and thermogravimetric analysis (TGA) (structural stability), as well as SEM (morphology) and transmission electron microscopy (TEM) are applied in characterizing ZIF materials. Diverse elemental analysis and spectroscopic methods have been employed in characterizing these new substances for determining their organic functional groups and confirming their chemical compositions [78].

#### 2.2.1. Fourier Transform Infrared Spectroscopy (FT-IR)

In their study, Jing et al. chose ZIF-8 as a photocatalyst for decomposing methylene blue (MB) under UV-light irradiation. According to the results, the peaks at 424 cm^−1^ (which could be attributable to the Zn–N stretch mode), 1565 cm^−1^ (C = N stretch mode), 2925 cm^−1^ and 3102 cm^−1^ (C–H stretch), and 995 cm^−1^ (which can be attributable to the C–N stretch mode) have been constant, which reveals the lack of changes in the chemical structure of ZIF-8 samples prior to and following photocatalytic reaction [79].

In addition, Xu et al.’s study analysed the rapid speciation of methylmercury and mercury for synthesizing ZIF-7 and ZIF-60. For additional characterization of the resulting MOF particles, researchers collected their FT-IR spectra. For Mim and benzimidazole (bim), imidazole (Im)—applied to synthesize ZIF-60 and ZIF-7, respectively—they have observed a sharp band at ~3125 cm^−1^ because of C–H stretching, a weak band at ~1820 cm^−1^, and numerous robust wide N–H bands between 3335 and 2500 cm^−1^. For the ZIF-60 and ZIF-7 spectra, each band disappears, revealing the correlation of bim, Im, and Mim with the ZIF-7 or ZIF-60 frameworks, which completely deprotonate [33].

#### 2.2.2. Powder X-ray Diffraction (PXRD)

XRD is the commonest method of analysing MOFs’ structures due to their periodic nature. Nevertheless, as the growth of a MOF crystal with constant proper quality and size is not feasible, researchers frequently utilize powder X-ray diffraction (PXRD) at room temperature. Therefore, PXRD patterns have shown the reproducibility of the synthesis results or explanations of the structural differences between the samples of the similar MOFs prepared by various techniques. Additionally, PXRD has proved the structural integrities of the frameworks. Hence, it has been applied in distinguishing the phase structures and structural integrities of the as-prepared samples [80,81].

In addition, Christus et al. have investigated the production of Fe_3_O_4_@ZIF-67 nanocomposite as a colorimetric determination of a Hg(II) sensor and have attributed the typical diffraction peaks of the Fe_3_O_4_@ZIF-67 nanocomposite at 18.20°, 30.6°, 35.9°, 43.2°, 53.8°, 57.6°, and 62.8° to the (111), (220), (311), (400), (422), (511), and (440) planes matching the Fe_3_O_4_ (JCPDS No. 19-0629) structure. According to their observations, the peaks of ZIF-67 at 7.39°, 10.36°, 12.79°, 14.71°, 16.45°, 18.01°, 22.19°, 24.64°, and 26.72° correspond to the (002), (112), (022), (013), (222), (114), (233), (044), and (134) planes of ZIF-67. Finally, XRD patterns have proved the creation of the Fe_3_O_4_@ZIF-67 nanocomposite and completely matched with the crystalline structure of ZIF-67 and the spinel structure of Fe_3_O_4_ [82].

Furthermore, Huo et al. use PXRD pattern analysis to characterize the Fe_3_O_4_@ZIF-8 composite and attributed the same diffraction peaks of the Fe_3_O_4_@ZIF-8 and Fe_3_O_4_ composites at 2θ = 35.1°, 42.9°, 56.7°, and 62.5° to (311), (400), (511), and (440) that are relatively compatible with the purity phase of Fe_3_O_4_ (JCPDS no.19-0629). Ultimately, other peaks, at 7.4° (011), 10.3° (002), 12.9° (112), and 18.0° (222) of Fe_3_O_4_@ZIF-8, have been accorded to those of ZIF-8 that correspond to earlier crystalline ZIF-8 [83].

#### 2.2.3. Field Emission Scanning Electron Microscopy (FESEM)

SEM is one of the most used tools for measuring the diverse features of MOFs, such as the size of the crystals, their elemental composition, and their morphologies. Based on the size of the MOF crystals, it is possible to use optical microscopy for obtaining valuable data on its morphology and size. SEM has been also used to examine the shape and size of ZIF particles [81,84].

In their study, Zhang et al. addressed the production of the Fe_3_O_4_@ZIF-8 magnetic cor@shell microspheres; to this end, they used SEM images and have shown the spherical shape of Fe_3_O_4_ particles, with an average diameter of ~600 nm. It is notable that, in the preparation of Fe_3_O_4_@ZIF-8 cor@shell microspheres, the essential phase has been to form a smooth and continual ZIF-8 shell on the Fe_3_O_4_ microspheres [85].

According to Zhao et al.’s study, ZIF-8/C_3_N_4_ composite, which has been considered an effective photocatalyst, may produce H_2_O_2_ with greater efficiency (2461 μmol·h^−1^·g^−1^) under visible light (λ ≥ 420 nm) and at a normal pressure without any considerable agents. Regarding SEM images, C_3_N_4_ possesses a sheet-like structure and ZIF-8 nanocrystals exhibit rhombic dodecahedra structures with an average size of ~100 nm. Moreover, the microstructure and morphology of ZCN (unspecified ZCN represents the composite synthesized by ZIF-8 and C_3_N_4_ with a mass ratio of 1:1.5, calcined at a temperature of 400 °C) with SEM has shown loading of ZIF-8 NPs on the C_3_N_4_ surface [86].

**Figure 2:**
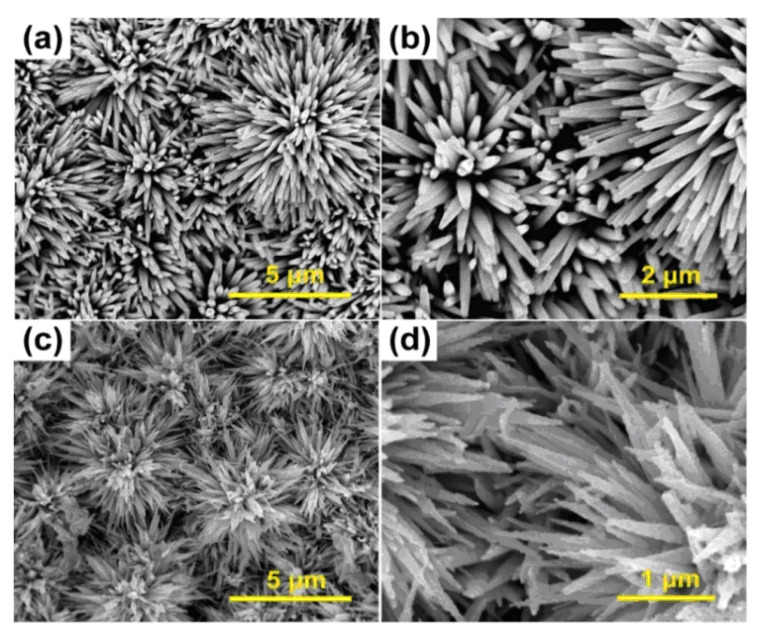
SEM images of WO_3_@ZIF-71 and WO_3_ nano-rods; (**a**,**b**) WO_3_ nano-rods at various magnifications; (**c**,**d**) WO_3_@ZIF-71 nano rods at various magnifications [87].

Zhou et al.’s study synthesizes cor@shell flower-like WO_3_@ZIF-71 via the pretreatment of WO_3_ with a ZnO precursor solution. They have found that it is possible to smoothly cover WO_3_ with ZIF-71. Figure 2 represents SEM images of WO_3_ and WO_3_@ZIF-71 nano-rods. Figure 2a depicts uniform WO_3_ nano-rods with a flower-like structure and Figure 2b is a magnified image thereof, wherein the nano-rods are shown to have a diameter of ~200 nm. According to the outputs, the morphology of the nano-rods has been modified following a reaction with ZIF-71. Considering Figure 2c,d, researchers have observed visible rough and dense coatings of ZIF-71 on WO_3_ nano-rods, with greater diameters, of ~300 nm, than those of pure WO_3_ nano-rods, demonstrating the successful assembly of the WO_3_@ZIF-71 cor@shell structure [87].

#### 2.2.4. Transmission Electron Microscopy (TEM)

TEM is largely used for determining the sizes of grains and particles, and crystallographic data such as dislocations and plane indices. Moreover, it is possible to use various software programs to analyse the microscopy images and identify the sizes of the particles and to measure various particles and the spacing between the crystallographic planes. Therefore, it may be highly beneficial to characterize MOFs modified by incorporating NPs, due to the data on dispersion and size of the NPs provided by the obtained images [88].

A study conducted by Liu et al. presents one of the new active tumour-targeting systems (Fe_3_O_4_@TiO_2_@ZIF8-DNM NPs) through daunomycin (DNM) as a model of an anti-tumour drug with cor@shell hybrid NPs (Fe_3_O_4_@TiO_2_@ZIF8 NPs) as the carrier. With regard to the microscopic images, researchers have shown the spherical morphology of the particles. Therefore, it has been possible to observe Cor@shell hybrid Fe_3_O_4_@TiO_2_@ZIF-8 NPs in the TEM images such that the core and external layers of the particles could be differentiated. As demonstrated by the TEM image of Fe_3_O_4_@TiO_2_@ZIF-8 particles, the average diameter of Fe_3_O_4_@TiO_2_@ZIF-8 particles is 400 nm [89].

Wang, et al. employ ZIF-67 as a precursor to synthesize several Co/NCs nano-cubes with special morphologies. According to TEM, dark dots with various dimensions have been not smoothly distributed in the carbon cube matrix and the size of the Co/NC-700 particles is nearly 300 nm. Actually, TEM illustrated the wrapping of Co/NC-700 NPs via layers of carbon sheets. The sheets have been created at higher temperatures, in situ, using Co as a catalyst. Finally, high-resolution TEM (HRTEM) has exhibited completely ordered crystalline lattices, in which the lattice fringes of both planes are nearly 0.205 nm apart, which corresponds to the (111) crystal plane of cubic Co/NC-700 [90].

Furthermore, Tian et al. show the successful synthesis of the ZnO@ZIF−8 nano-rods using cor@shell hetero-structures under hydrothermal conditions at a temperature of 70 °C for 24 h. They consider the outer shell, shown with light contrast, to be the ZIF−8 layer with a uniform coating on the surface of the internal ZnO nano-rods, shown with dark contrast. The ZnO core diameter is nearly 300 ± 50 nm, whereas the surface of the ZnO nano-rods appears rough and deposited with a ZIF−8 shell of 100 ± 50 nm. However, for ZIF−8’s structure, coordination of Zn-atoms’ centres by N atoms in the 1,3-positions of the five-membered ring has been observed and neutral frameworks have been constructed on the basis of nets of the linked ZnN_4_ tetrahedra [91].

#### 2.2.5. Thermogravimetric Analysis (TGA)

TGA has been proposed as one of the widespread techniques for determining MOFs’ thermal stability and estimating the respective solvent-accessible pore volume. As measurement of MOFs’ thermal stability, considering the carrier gas species (such as air, O_2_, and N_2_) selected with which to measure would be of high importance, it therefore must be constantly specified, due to the possible variability in the decomposition pathways of MOF in various environments. Additionally, identifying MOF thermal stability through TGA measurements must be considered because the mass loss maybe not inevitably correlated to structural changes [84]. Therefore, nitrogen has been used as the carrier gas to implement TGA on ZIFs and the heating rate has been fixed to 2 K min^−1^ in ranges between 30 °C and 700 °C [92].

In their study, Wu et al. address the synthesis of a ZnO@ZIF-8 cor@shell nanorod film as a gas sensor with very good selectivity for H_2_ over CO. They examine the stability of a ZnO@ZIF-8 cor@shell nanorod sensor for functional uses and employ TGA for characterizing its thermal and structural stabilities. According to them, their ZnO@ZIF-8 cor@shell nanorod film exhibits up to 320 °C stability, resulting in the complete coverage of its optimal working temperature ranges (200 °C to 250 °C) as an H_2_ gas sensor. The researchers have also measured its capability for moisture endurance and have demonstrated an acceptable capability of their ZnO@ZIF-8 cor@shell nanorod sensor to endure moisture [93].

**Figure 3:**
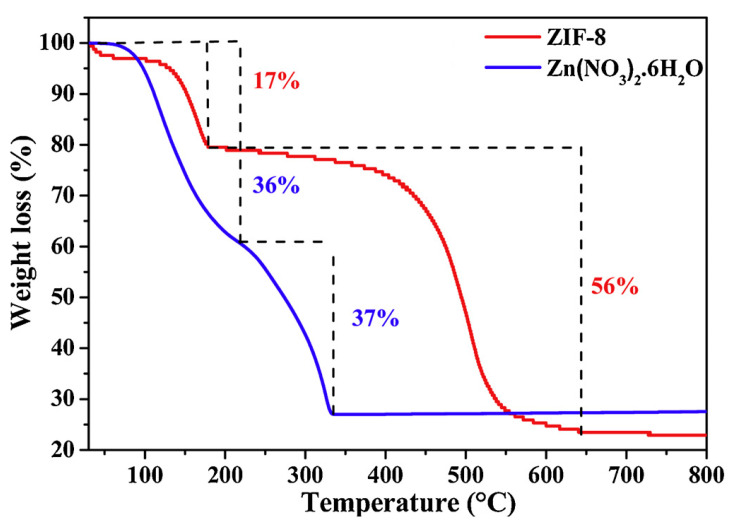
Comparison of TGA profile of semi-conducting ZnO synthesized with a ZIF-8 template by pyrolysis (blue curve) and with ZIF-8 (red curve) [94].

Moreover, Payra et al. created semi-conducting ZnO via ZIF-8 template pyrolysis. They screened the materials for the electro- and photocatalytic oxidation of methylene blue and a TGA profile of ZIF-8 shows it exhibits nearly 20% weight loss at decreased temperatures (<200 °C), which may be caused by the weight losses at lower temperature ranges due to the elimination of the inorganic (NH_4_^+^) guest. Notably, weight loss is nearly 60% at greater temperatures (nearly 650 °C). Hence, the researchers illustrate the correspondence of weight loss with the combustion of the organic frameworks and the creation of ZnO at greater temperatures (450 °C to 650 °C). Thus, regarding their TGA profile, they calcine ZIF-8 in the air at a temperature of 450 °C for two hours to achieve a synthesized ZnO (ZIF). The TGA profile of Zn(NO_3_)_2_.6H_2_O exhibits nearly 36% weight loss by 200 °C as a result of the elimination of six water molecules in the salt and, consequently, a second weight loss of 37% at the greater temperature of 350 °C may correspond to nitrate decomposition in forming stable ZnO. Finally, the researchers have not observed any weight loss at further increased temperatures, which implies the high thermal stability of ZnO (Figure 3) [94].

#### 2.2.6. N_2_ Adsorption–Desorption Isotherms

As mentioned in different studies, surface area has been proposed as one of the prominent parameters for optimizing materials for several uses, such as gas storage. Although Brunauer–Emmett–Teller (BET) has extensive uses in assessing mesoporous and microporous adsorbents, in case of application in the classical range of monolayers and starting multilayer formation (P/P_0_ = 0.05–0.3), it can be utilized for macroporous, mesoporous, and nonporous substances with pore widths greater than 4 nm [95]. Nonetheless, researchers have found that the mechanism achieved by BET cannot be utilized for microporous substances and, therefore, the computed area for micropores consisting of adsorbents must be examined as a “BET area”. Finally, exact criteria must be employed for determining the linear ranges when calculating BET. The above criteria have been defined in the recent recommendations by International Union of Pure and Applied Chemistry (IUPAC) [95,96].

To this end, Venna et al.’s investigation concerning the structural evolution of ZIF-8 shows type-I behaviour of the nitrogen adsorption–desorption isotherms that are characteristic of microporous phases. Their results have also shown the correlation of the specific surface area of BET to ZIF-8’s relative crystallinity [97].

Consequently, Huang et al.’s study reports the synthesis of zinc(II) imidazolates and determined the specific surface area using the BET method, as well as relative pore size distribution. They have found that their stability can be compared with that of the most thermo-stable porous MOFs. The researchers also consider the N_2_ sorption features of Zn(Eim)_2_·H_2_O, “2-Ethylimidazole (Eim)”, and Zn-(Mim)_2_·2H_2_O. Finally, their Zn-(Mim)_2_·2H_2_O compound obtains a typical type-I isotherm with a Langmuir surface area of 1400 m^2^g^−1^ (BET surface area of 1030 m^2^g^−1^) [98].

## 3. Applications

ZIF materials have been largely used due to their higher porosity and tunable compositions and structures. Researchers have introduced pure ZIFs and ZIF-based materials as multifunctional ones that show flexible, good function beyond their conventional applications as catalysts and adsorbents because they can be used in areas such as electrochemistry, electrical equipment, and drug delivery. Therefore, new uses of ZIF materials will be presented here [10].

The greater moisture and thermal stability of ZIFs in comparison with other MOFs [99] and their greater cavities than those of corresponding zeolites are the results of the robust and longer bonding among their metal centres and the imidazole centres [100,101]; hence, researchers have extensively investigated the exceptional features and uses of ZIFs [102].

According to the research, specific surface area, the size of cavity due to the interconnected ZIF particles, and the pore diameters of ZIF have been considered as the efficient variables in the function of porous ZIFs. For this reason, researchers have considered ZIF-8, with an adsorption capacity of up to 3000 m^2^/g and higher surface areas of 1408 m^2^/g and 1384.2 m^2^/g, more than the other ZIF structures [103].

### 3.1. Gas Separation

Researchers have also employed MOFs consisting of considerable micropores as flexible building blocks for energy storage equipment and catalytic systems and, notably, they have been increasingly attracted by MOFs’ potential as molecular sieves for chemical sensors [104].

Numerous researchers have examined ZIF materials as gas separation membranes or fillers for mixed-matrix membranes in comparison with other MOFs, due to their higher thermal and chemical stabilities and specific framework flexibility [105].

In their study, Pan et al. show the synthesis of ZIF-8 membranes in aqueous solution on porous α-alumina discs using a new hydrothermal seeded growth procedure. Some researchers have also employed ZIF-8 membranes to determine the separation performance of C2/C3 hydrocarbon mixtures and have observed very good separation performance therewith that has been not observed in any other membranes. Table 1 reports the effective separation of binary mixtures, such as H_2_/C_3_H_6_, H_2_/C_3_H_8_, C_2_H_4_/C_3_H_6_, C_2_H_4_/C_3_H_8_, and C_2_H_6_/C_3_H_8_ by our new ZIF-8 membrane. Additionally, the permeabilities of H_2_ in the H_2_/C_3_H_8_ mixture, C_2_H_6_ in the C_2_H_6_/C_3_H_8_ mixture, and C_2_H_4_ in the C_2_H_4_/C_3_H_8_ mixture, calculated from Table 1, are around 3600 GPU, 600 GPU, and 1200 GPU, respectively. The penetration of all gases in mixed gas systems is similar to that of single gas systems, and the separation factors of the gases are close to their ideal selectivity. All of these gas mixtures can be found extensively in processes such as natural gas processing, oil refining, and chemical processes. For example, hydrocarbons C1 to C4 are all important raw materials in the petrochemical industry.

They are currently produced mainly by separation from natural gas through cryogenic distillation, which requires a lot of capital and energy. Therefore, the ZIF-8 membrane developed in this connection must have great potential to provide an energy-efficient process and low cost in separating these mixtures [106].

Li et al.’s study addresses the production of ZIF-8 tubular membranes through a recyclable 2-methylimidazole water–solvent solution by self-converted ZnO nano-rods. According to their analyses, the self-converted synthesis uses a ~0.005 M ligand to obtain a continuous ZIF-8 membrane. For this reason, it is possible to iteratively utilize a solution with a ligand concentration of 0.5 M for not fewer than 50 times to prepare compact ZIF-8 membranes without treatment. This membrane may yield H_2_ permeances of nearly 1.1 × 10^−7^ mol·m^−2^·s^−1^·Pa^−1^ with an optimal selectivity of >40 for H_2_/CH_4_ and very good operating stability [107].

Additionally, Lo and Kang deal with the synthesis of hybrid ZIFs membranes with greater separation functionality by a method that requires pseudo-polymorphic seeds. According to them, ZIF-L@ZIF-8 hybrid membranes have greater molecular permeability for N_2_, H_2_, CO_2,_ and CH_4_, and enhance permeative selectivity for H_2_ over CO_2_, from 2.3 to 4.7, in comparison with pure ZIF-8 membranes. The researchers also demonstrated that the interlayer spacing among ZIF-L crystals, which rapidly diffuses H_2_, maybe the main cause of the high separation functionality of ZIF-L@ZIF-8 hybrid membranes [108].

### 3.2. Electrosynthesis

**Figure 4:**
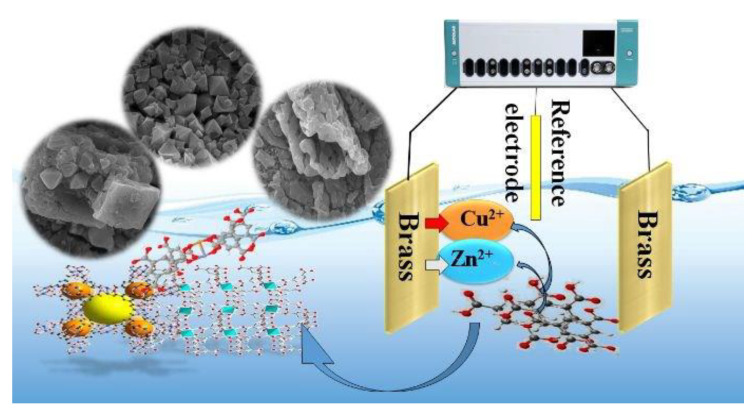
Simultaneous electrosynthesis of MOFs on brass by the electrochemical technique; by increasing the potential from 1 to 10 volts, different morphologies and smaller crystals are created [110].

Furthermore, electrochemical synthesis enjoys numerous merits: (1) more rapid synthesis at declining temperatures in comparison with conventional synthesis; (2) it is not necessary to have metal salts, hence, separating the anions, such as Cl^−^ and NO_3_^−^, from the synthesis solution would not be needed; and (3) it is possible to achieve the total use of the linker [109]. The fundamental synthesis principle requires the supply of the metal ion through anodic dissolution to a substrate mixture consisting of electrolyte and organic linker. Nevertheless, this procedure releases H_2_ in the course of the electrochemical synthesis, derived from the dissolved molecules of the organic linker [78]. Figure 4 depicts the simultaneous synthesis of Cu–BTC/Zn–BTC MOF films on a brass alloy with the help of the electrochemical technique at room temperature in a shortened time [110].

**Figure 5:**
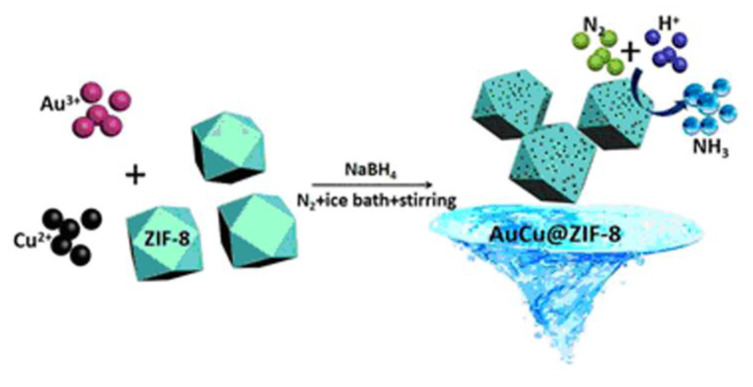
Schematic synthesis of AuCu/ZIF-8 by a one-step synthetic path for obtaining the electrochemical synthesis of ammonia [111].

Furthering this, Lv et al.’s study employs a gold-copper nanoalloy-decorated ZIF, with the name AuCu/ZIF-8, in obtaining the electrochemical synthesis of ammonia (Figure 5). The above composite has been proposed to be a pH-independent, high performance stable electrocatalyst for nitrogen reduction reactions (NRR) in nitrogen-saturated electrolytes. Actually, in an acidic electrolyte with the H_2_ content of air, AuCu/ZIF-8 would achieve an unexpected ammonia production rate of 23.3 μg h^−1^ mgcat^−1^ amongst all electrocatalysts, which refers to one of the significant steps in industrializing green ammonia [111].

### 3.3. Electrochemical Performance

In their study, Ajdari et al. provide a polymer/ZIF-67 nanocomposite film using the electrochemical deposition technique. They show greater charge storage capacity and electrochemical activity of their composite films. The researchers have shown a significant improvement of ZIF-67, resulting in more active sites on the composites for Faradaic reactions and a greater specific capacitance than has pure POAP. The above result shows higher electrical conductivity, decreasing resistance, and simplifies the composite charge transfer. Ehsani et al. attributed the supercapacitive behaviour of the composite films to the higher active surface areas of the composites, to charge transfer alongside the polymer chain because of the polymer conjugation form, and to synergistic effects of the conductive polymer with ZIF-67 [112].

### 3.4. Gas Sensor

Zhang et al.’s study addresses the synthesis of ZIF-8 and ZIF-90 using the bio-mimetic method in an aqueous solution and test the gas-sensing capacity of quartz crystal micro-balance (QCM) transducers based on ZIFs. According to their experiments, ZIF-90-based sensors enjoy very good sensing function at lower concentrations of acetone vapor, such as higher sensitivity (a frequency shift of 95 Hz @ 1 ppm), faster response (response/recovery time of 12 s/17 s), and acceptable selectivity. With regard to the temperature-varying experimental technique, a moderate adsorption enthalpy (ΔH^θ^ value equaled −57.93 kJ/mol) has been observed that implies the existence of weak and reversible chemisorption between ZIF-90 and acetone molecules. Finally, the QCM sensor, based on ZIF-90, in comparison with ZIF-8 has very sensitive performance toward low concentrations of acetone vapour [113].

### 3.5. Anti-Bacterial and Anti-Microbial

Another study conducted by Ahmad et al. presented a fast procedure for synthesizing ZIF-8-decorated graphene oxide (GO) composites (ZGO) with acceptable anti-bacterial features. According to the characterization outputs, the researchers have observed a successful decoration of ZIF-8 with a size of ~120 nm on the surface of the GO sheets with the host ZIF-8 framework preserved in the synthesized composite. Then, they use minimum inhibitory concentration (MIC) tests and disc diffusion for determining the anti-bacterial activity of the samples against Staphylococcus aureus ATCC 6538 and Escherichia coli ATCC 11,229 as the model strains of gram-positive and -negative bacteria. Finally, ZGO-1.0 (1 wt% of the GO-to-metal salt ratio) shows the greatest anti-bacterial activities, with the MIC values crucial for the inhibition of the bacterial growth of E [114].

Furthermore, Nabipour et al. present ZIFs with ciprofloxacin (CIP) at the nano-scale. They synthesize porous ZIFs in a concentrated aqueous solution and utilize a simplified nano-precipitating technique for preparing CIP-loaded ZIF-8 (CIP-ZIF-8). In their analyses, the researchers observed the successful encapsulation of antibiotic drugs in ZIF- 8 with increased loadings (21 wt%). Moreover, ZIF-8 exhibits a higher loading capacity for the model antibiotic drug, CIP, with a pH-sensitive release. Results have also shown that the as-synthesized CIP-ZIF-8 enjoys higher drug-loading efficiency (21%), and release in pH-5.0 acetate buffer solutions (97%) is more rapid than in pH-7.4 phosphate buffers (PBs) (83%). They report an increased potential for the use of MOF for diverse bio-medical uses and drug delivery systems on the basis of nano-materials. Finally, the anti-bacterial activities of CIP-ZIF-8 have been investigated using diffusion disc assays, which indicate the greater anti-bacterial activities of CIP-ZIF-8 than CIP and ZIF-8 [115].

### 3.6. Protection of Proteins

According to Poddar et al.’s study, an efficient method to protect proteins would create biomolecule–MOF composites; therefore, they have examined the encapsulation of a full gene-set in ZIF-8 MOFs and the cellular expression of the genes delivered by nano–MOF composites. Then, they show the maintenance of the functional activities of the encapsulated genes, which indicates intracellular delivery. It has been also found that using water-washed plGFP@ZIF-8 for gene delivery and cellular expression may influence efficiency and uptake [116]. 

### 3.7. Drug Release and Delivery

Since ZIF materials have very good porous structures, specific chemical and thermal stability, and adjustable multi-functionality in their frameworks, as well as pH-sensitive release characteristics, they have been considered as robust platforms for the controlled release of drug molecules and for drug delivery [117].

Consequently, Vahed et al.’s study addresses the synthesis of an alginate-coated ZIF-8 combination as a green and bioactive platform, investigating its uses for the controlled release of drugs. They show the stability of coating in acidic environments that results in the targeted release of drugs in non-acidic media, such as the intestine. Concerning the extraordinary features of coated ZIF-8 in high-pH environments, this material could be proposed as an excellent candidate for the delivery of drug agents, in particular, of acid-sensitive and non-anticancer medicines. However, if making a comparison with other cor@shell DDSs, these cor@shell NPs exhibit certain benefits. In fact, coated ZIF-8 NPs do not have any toxicity to normal cells at various pH conditions and have been capable of releasing their cargos in high-pH media. Therefore, the presence of zinc ions and the regular porosity of the DDS may be beneficial in increasing the efficiency and controlled release of drugs (i.e., metformin) [118].

Another research effort, conducted by Ran et al., presents hollow poly(dopamine) (PDA) nanocapsules using the aqueous one-pot synthesis procedure, with the help of ZIF-8 nanocrystals as a sacrificial template without any specific etchant. The researchers have observed minor cytotoxicity in the resulting PDA nanocapsules to HeLa cells after incubation at different doses for 48 h, demonstrating their potential as an alternative for functional uses in the targeting and transferring of drugs [119].

### 3.8. Adsorption in Aqueous Solution

As mentioned earlier, MOFs have been considered as novel alternatives for gas separation and gas-adsorbent tools. Researchers have addressed the adsorption function of zeolites and their ZIFs for diverse gases, such as CO_2_, N_2_, O_2_, etc., and have used computational screening for their comparison. Based on the findings, no association exists between the adsorption heat values of zeolites and their ZIFs [120].

A study conducted by Abdelhamid and Zou employ a novel approach to synthesizing hierarchical porous ZIF-8 NPs in aqueous solutions at room temperature, in which they obtain by introducing sodium hydroxide into the zinc nitrate solution before the addition of the linker Mim to the reaction mixture. They show that ZIF-8 and ZIF-L (ZIF leaf-like materials) provided by the above approach would have acceptable CO_2_ sorption features. The ZIF-8 NPs exhibit rapid (<5 min), selective, and high-efficiency (>95%) uptake of methyl blue in aqueous solution without and in the presence of other dyes [121].

Another investigation, by Taheri et al., addresses the conversion of ZnO nanopowder to ZIF-8 using a green method through a stoichiometric mechanochemical reaction. They show the specific features of the obtained NPs appropriate for water remediation uses. In fact, using a small content of methanol in liquid-assisted grinding (LAG) has been considered crucial in breaking the agglomeration of ZnO NPs, as well as in the complete conversion of ZnO to ZIF-8 NPs. Their findings have also shown a higher surface-to-volume ratio and more adsorption sites for model organic pollutants than ZIF-8 produced from a conventional material by mechanochemically synthesized ZIF-8 NPs. Therefore, mechanochemically synthesized ZIF-8 has been proposed as one of the major materials for eliminating pollutants [122].

In their study, Jaberi et al. have aimed at the conversion of cigarette filters (CFs) to cigarette carbonaceous (CC) hydrochar as a green and inexpensive approach for producing a new adsorbent with lower toxicity and added value in CO_2_ capturing. Therefore, they employ a five-level CCD-based RSM to investigate the role played by four key operational variables (liquid flow rate, gas flow rate, CO_2_ initial concentration, and CC-hydrochar/ZIF-8 MOF loading) of CO_2_ capture efficiency. They have observed the greatest CO_2_ capture efficiency, of 99.15%, at the liquid flow rate, gas flow rate, CO_2_ initial concentration, and CC-hydrochar/ZIF-8 MOF loading of 0.40 L/min, 3.30 m^3^/h, 3000 ppm, and 0.06 g/L, respectively. They have found that optimizing operation conditions could elevate the CO_2_ capture efficiency of CC-hydrochar/ZIF-8 MOF-based fluid by 32.36% in comparison with the bare 30 wt% MEA solution. Hence, the presence of a little amount of CC-hydrochar/ZIF-8 MOF in MEA-base fluids may considerably elevate the CO_2_-capture efficiency associated with the acceptable features of CC-hydrochar/ZIF-8 MOF (higher surface area, more functional groups, inter-connected wall structure, smaller pores, and larger pore volume) [123].

Moreover, the development of ZIF-67 hydrocarbon/liquid carbon cigarettes for CO_2_ absorption from gas flow in an aqueous medium has been reported in Jaberi et al.’s study. Their data reports the highest CO_2_ capture percentage (98.02%) under optimal conditions of a liquid flow rate of 0.40 L/min, a CC-hydrochar/ZIF-67 loading of 0.06 g/L, a CO_2_ initial concentration of 4500 ppm, and a gas flow rate of 3.00 m^3^/h. Then, the researchers assess the mass-transfer performance in the applied absorbents via computing the overall mass-transfer coefficient in each unit volume (KGa), the number of transfer units (NTU), the total height of packing in the column (Z), and the height of transfer unit (HTU). Finally, they propose CC-hydrochar-ZIF-67 nano-fluid as an effective and affordable alternative to conventional absorbents [124].

In their study, Pan et al. address the preparation of a novel group of N-doped hierarchical porous carbons with higher surface areas and CO_2_/N_2_ selectivity and impressive CO_2_ capacities with a new mixed-ligand ZIF (zeolitic imidazolate framework) (JUC-160) as the precursor. They identify the resulting samples as mJUC160-900, mJUC160-1000, and mJUC160-1100, and nJUC160-900, nJUC160-1000, and nJUC160-1100, which, respectively, correspond to carbonization temperatures of 900 °C, 1000 °C, and 1100 °C. Their findings indicate the best CO_2_ uptake of 5.50 and 3.50 mmol g^−1^ at 273 and 298 K by the mJUC160-900 material, and achieve a high-adsorption selectivity for CO_2_/N_2_ at 1 bar and 298 K. Such an exceptional function is caused by higher concentrations of nitrogen accompanied by desirable microporosity. Therefore, it may be one of the efficacious adsorbents in capturing flue-gas CO_2_ [125].

### 3.9. Catalyst

Since ZIFs have been proposed to be analogous to the other kinds of porous, aluminosilicate zeolite materials, researchers have addressed major and commercially available catalytic materials—ZIF-based materials and ZIFs—as effective catalysts for several reactions, even though these must be further developed. In fact, pure ZIF substances may be active catalysts for several reactions, such as trans-esterification, FriedelCrafis acylation, the Knoevenagel reaction, the synthesis of carbonate and mono-glyceride, epoxidation, and hydrogen production and oxidation. Experts in the field have illustrated ZIFs, such as ZIF-9, ZIF-10, and ZIF-8, as effective heterogeneous catalysts for the Knoevenagel reaction between malononitrile and benzaldehyde condensation for forming benzylidene malononitrile. Therefore, it is possible to separate ZIF catalysts from reaction mixtures and to reapply them with no considerable degradation in their catalytic activities [10].

Additionally, Bhin et al. deal with the catalytic potential of a chloro-functionalized ZIF-95 for the solventless cycloaddition of CO_2_ with epoxides. They optimize the reaction variables and choose a moderate set of reaction conditions (1.2 MPa CO_2_ pressure and 80 °C) to completely investigate the catalytic process. It is notable that the researchers show the catalytical activation of ZIF-95 at greater temperatures, but adding a co-catalyst helped the reduction in temperature that is necessary for achieving high substrate conversions. As a next step, Park et al. have used a binary catalyst system of ZIF-95/TBAB (tetra-n-butylammonium bromide) to achieve 83.2% conversion of propylene oxide (PO) with ~99% selectivity to the intended product; that is, propylene carbonate (PC) under similar moderate conditions. Results have shown the chemical stability of the catalyst system and exposure to reuse four times without any remarkable decline in catalytic activity [126].

**Figure 6:**
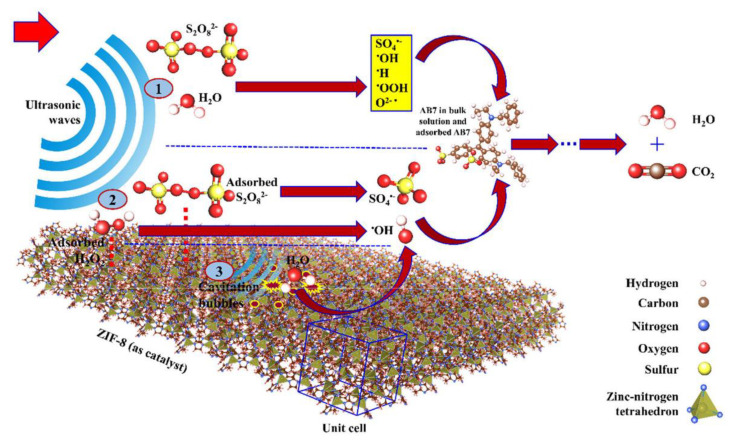
Heterogeneous and homogeneous decomposition mechanism for H_2_O and PDS molecules on a catalyst surface and bulk solution, causing the constant creation of active oxidizing samples (paths (1) to (3)) [127].

Furthermore, Sisi et al. have addressed the systematic activation of potassium peroxydisulfate (PDS) with ZIF-8 using a sono-assisted catalytic process, employing a combination of a sonolysis process and ZIF-8 nanomaterials (NMs) in order to efficiently activate PDS and have established a new combined sono-assisted indirect photocatalytic process. They also have determined the impact of the operational variables, such as ZIF-8 content, PDS content, pH, ultrasonic bath power (UBP), and Acid Blue 7 (AB7) concentration, on three response parameters of the pseudo-first-order kinetic rate constant (kapp), synergistic factor (SF), and AB7 removal efficiency (RE (%)). They subsequently show the optimal operating conditions to be [ZIF-8] = 0.6 g/L, pH = 5, and [PDS] = 0.6 mmol/L with a maximum SF of 1.45. Due to the indirect activation of ZIF-8 NMs via the sono-luminescence phenomenon, the interference of diverse species, such as S_2_O_8_^2−^, HSO_5_^−^, and H_2_O_2_, has been observed in radical chain degradation reactions, which additionally create SO_4_^•−^ and ^•^OH radicals that elevate SF and AB7 RE (Figure 6). The researchers also apply GC–MS to detect the intermediates and recommend a degradation mechanism with regard to GC–MS output. Finally, ZIF-8 NMs indicate non-ecotoxic impacts, so the sono-catalytic process can promote the overall ecotoxicity reduction of AB7 in the course of a 90-min process [127].

**Figure 7:**
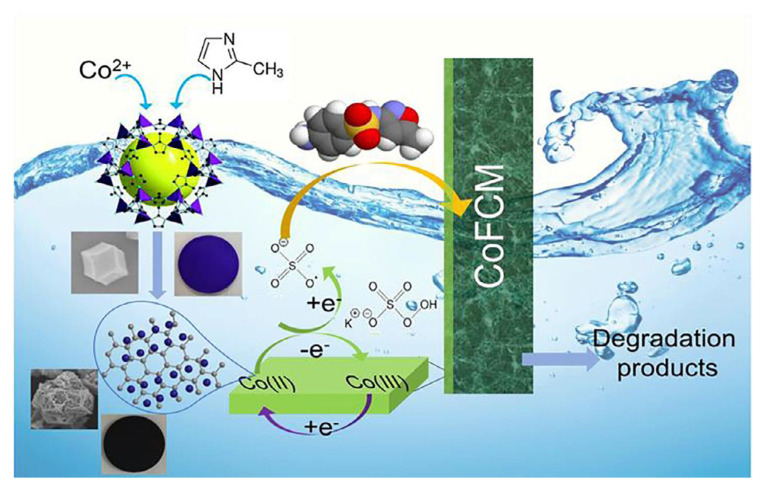
A new reaction mechanism for the degradation of SMX on the surface of ceramic membrane CoFCM [128].

Consequently, Bao et al. have created a Co-based ZIF; that is, ZIF-67 on the surface of the ceramic membrane (CoFCM) through surface-nucleated growth. They have tested the function of the resulting CoFCM in a home-made dead-end membrane filtration system and assessed the PMS activation mechanism in the CoFCM/Oxone system using an electron paramagnetic resonance (EPR) experiment and radical trapping. Their findings demonstrate the rougher surface of CoFCM with an initial membrane resistance of 1.19 × 10^11^ m^−1^, which performs a considerable catalytic activity. According to their findings, the pure water permeability of CoFCM is 3024 L m^−2^ h^−1^ bar^−1^, which could be compared to the pure permeability of the pristine ceramic membrane (<10% difference). In their next step, they obtain a sulfamethoxazole (SMX) removal efficiency of >90% in 90 min with the Oxone addition of 0.1 g L^−1^, such that the membrane exhibits very good durability by maintaining >95% of the initial flux for not less than three operational cycles with a low-cobalt ion leaching through Oxone-assisted cleaning. Finally, CoFCM shows increased performance in SMX removal and increased durability through Oxone-assisted cleaning [128] (Figure 7).

Another study, conducted by Azad et al., addresses synthesizing and characterizing an NHC ligand with a negative charge using a novel technique for stabilizing and controlling the aggregation of the small palladium NPs (Pd NPs) via ZIF-8 pores. Since negatively charged NHC enjoy electrostatic interactions, the functionalized Pd demonstrates a very suitable environment for the stabilization of Pd NPs, which subsequently prevents their unfavorable agglomeration. The researchers synthesized a hybrid nano-porous material; that is, Pd NPs@N-heterocyclic carbene@ZIF-8 and obtain a high-internal surface area for the successful preparation of a dispersed anionic sulfonated N-heterocyclic carbene–Pd(II) precursor in the cavity of ZIF-8 by the use of an impregnation procedure accompanied by reduction with NaBH_4_. They also employ compound materials for catalysing the Mizoroki–Heck cross-coupling reaction and, thus, it is possible to recover and reuse the catalyst not less than six times without considerable losses in its selectivity and activities [129].

Similarly, Kuruppathparambil et al.’s study deals with the synthesis of an environmental-friendly ZIF-67 for catalysing CO_2_ cyclo-addition and epoxides, which form cyclic carbonates in high yields with ~100% atom economy under solvent-free and co-catalyst-free conditions, whereas Zn consisting of ZIF-8 has been more reactive toward epoxide CO_2_ reactions. Results have shown the more acceptable selectivity of Co consisting of ZIF-67. It has been found that ZIF-67 can be thoroughly reused without any additional activation phases and, according to the ICP-OES analysis of filtrates, indicates a very small percentage (<1%) of leaching to the catalyst under conditions that show the maximum conversion rate of the epoxide. Analyses have also represented a certain acido-basicity of the structural defects on the exterior surface of the catalyst that is a rational result for the catalytic activities demonstrated by ZIF-67, and, generally, by each ZIF material [130].

### 3.10. Photocatalyst

As stated in several investigations [131,132], researchers have been largely attracted by numerous ZIF substances, which are employed as photocatalysts with visible light because of their universal higher demands for energy. An example is MOZIF-1, with a structure consisting of active MoO_4_ tetrahedral sites that results in their suitability for photocatalysis. Researchers have also examined Co-ZIF-9 as a photocatalyst in the reduction reaction of CO_2_ with the use of visible light at mild conditions. Nevertheless, the catalytic mechanisms of the MOF-mediated photo-catalysis cannot be clearly understood because photocatalysis is a charge transfer event following photo-excitation and, naturally, the charge transfer features of the structures of ZIF-derived photocatalysts remained to be addressed [120,132].

Another study, published by Chakraborty et al., achieves hetero-structure nanocomposites by integrating cubic-shape broad-bandgap ZIF-8 with narrower-bandgap CuO NPs. It has been found that recombination of the photogenerated e^−^/h^+^ pairs is promoted by increasing the bandgap in the CuO content of ZIF-8. The researchers choose CuO NPs/ZIF-8 material as a photocatalyst for decomposition of the rhodamine 6G (Rh6G) dye. Then, they deposit, with very small colloidal CuO NPs (nearly 5 nm), a ZIF-8 photocatalyst, indicating its effective photocatalytic activities to degrade Rh6G under sunlight irradiation. According to the results, 5 wt% CuO NPs deposit ZIF-8, showing the greatest photocatalytic activities, with a ~96% Rh6G dye removal efficiency, after 105 min of sunlight irradiation following the pseudo first-order kinetics model. The researchers have also shown the effective working of the catalyst in wider ranges of pH, from 5.0 to 12.0, and greater degradation efficiency in alkaline environments. Rapid decomposition of Rh6G dye, promoted by ROS species, has been also observed by adding an H_2_O_2_ solution to the reaction medium. Finally, it would be possible to use the nanocomposites as a strongly effective photocatalyst for decomposing organic pollutants [133].

Mahmoodi et al. have attempted, for the production of a ZIF-8/inorganic nanofiber (Fe_2_O_3_) nanocomposite, to use a green method of light-emitting diode (LED) irradiation for investigating photocatalytic degradation. Therefore, they fabricate ZIF-8/Fe_2_O_3_ composite nanofibers (ZFCN) with various contents of Fe_2_O_3_ nanofibers (5, 10, and 20 wt% defined as ZFCN-5, ZFCN-10, and ZFCN-20, respectively) under atmospheric pressure and room temperature. These researchers employ nanocomposites for studying the RR198 photocatalytic degradation, as compared with the α–Fe_2_O_3_ and ZIF-8 nanofibers and observe photo-degradation percentages of 52%, 75%, 41%, 84%, and 94% by ZIF-8, FN, ZFCN-5, ZFCN-10, and ZFCN-20, respectively. At the end, they find that RR198 photo-degradation follows the first-order model for ZFCN- 10, ZFCN-20, and ZFCN-5, as do the photocatalysts and the zero-order kinetics for α–Fe_2_O_3_ and ZIF-8 nanofibers [134].

In addition, Hassan et al.’s study deals with NP zinc oxide (ZnO) by calcinating ZIF-8 to remove the remazole red (RR) and acid red 57 (AR57) dyes at different conditions. Therefore, ZIF-8 has been calcinated at various temperatures of 450, 550, and 650 °C for synthesizing ZnO samples. The researchers have shown the strong dependence of the adsorption of anionic dyes on contact time, pH, and temperature. According to experimental outputs, the adsorption ability of ZnO for RR and AR57 are lower in basic rather than acidic solutions. They also reach the greatest removal at pH 2 and 3 for AR57 and RR, respectively, and the dyes’ adsorption is shown endothermic, because of the increased dye removal capacity from increasing temperature via the enhanced mobility of the dye molecules. Hence, the pseudo-second-order kinetic model thoroughly matches the dynamic behaviours of absorbing RR and AR57 towards ZnO. Finally, El-Bindary et al. have achieved adsorption efficiencies of ZnO NPs on the order 450 > 550 > 650 °C [135].

### 3.11. Removal Efficiency

In their study, Samadi-Maybodi and Rahmati address ZIFs’ synthesis with Co^2+^ and Zn^2+^ and use cefixime as an adsorbate under the same conditions for comparing the removal efficiency of two-metal and single-metal ZIFs. They show that the existence of cobalt promotes the physicochemical features, such as crystal structure, specific area, and magnetic properties, as well as porosity, and maintains ZIFs’ basic structures. Then, the researchers examine the removal efficiency for the two NPs at optimal conditions, which is provided by their BOX–Behnken design. Their results show the stability of the composite at various pH values and the greater sorption capability of ZIF-2M (97%) than that of ZIF-8 (92%) [136].

Jafari’s study addresses the production of ZIF-8 with two environmental-friendly and affordable procedures in an aqueous solution at room temperature. There, the researchers experiment with ZIF-8 samples activated thermally (under a dry air atmosphere) and under vacuum for evaluating the adsorption effectiveness in extracting toluene and carbon tetrachloride (typical VOC molecules) from the polluted air stream in a continuous (dynamic) system. Then, he applies a breakthrough curve for determining the impact of various pre-treatment conditions, the size of the particles, and surface areas of ZIF-8 on the performance of adsorption. With regard to the experimental outputs, adsorption performance ZIF-8 is ameliorated by enhancing the pre-treatment temperature and reducing the size of the particles. It is notable that results of the experimental dynamic adsorption of toluene and carbon tetrachloride on ZIF-8 matched with Thomas’ and Yan’s models and that the breakthrough curves of the toluene adsorption matched the two models better than the curves of carbon tetrachloride [137].

Subsequently, Cao et al. provide magnetic ZIF-67/GO composites through a one-pot method at room temperature to extract neonicotinoid insecticides from environmental water samples. They initially employed the synthesized materials as adsorbents in magnetic solid-phase extraction (MSPE) to extract neonicotinoid insecticides. Then, the researchers optimize the key experimental variables, such as the amount of the added magnetic composites, ionic strength, and desorption solvent, as well as extraction pH, to increase the absorbance capacity for neonicotinoid insecticides. According to the findings, the limit of detection (LOD) ranges from 0.06 to 1.0 ng/mL based on the optimum conditions. Moreover, the analytes present an acceptable linearity with the correlation coefficients of >0.9915, and the relative standard deviations (RSDs) for five neonicotinoid insecticides in the environmental samples are in the range of 1.8 to 16.5%, with reasonable recoveries between 83.5 and 117.0%, which demonstrates the feasibility of magnetic ZIF-67/graphene oxide composites in analysing trace analytes in environmental water samples [138].

In their study, Huang et al. devise a simplified, reliable, and sensitive analytical method with regard to an IL-modified magnetic multi-walled carbon nanotubes (MWCNTs)/ZIF-8) composite as an adsorbent for MSPE of the dichlorodiphenyltrichloroethanes (DDTs) from environmental water samples. They also present a magnetic IL adsorbent based on a modified magnetic multiwalled carbon nanotube, MM/ZIF-8/IL, which has been synthesized via the immobilization of IL on the MM/ZIF-8 surface. This new composite is a combination of the benefits of magnetic MOFs, IL, and MWCNTs that have shown reasonable selectivity and acceptable adsorption capability. The researchers have also addressed the optimization of multiple experimental conditions influencing MSPE efficiency and employed the prepared MM/ZIF-8/IL as an adsorbent for MSPE of DDTs from environmental water samples. In combination with GC-MS/MS, the MM/ZIF-8/IL-based procedure offers a wider linear range, very good sensitivity, and acceptable accuracy. As compared with other techniques proposed to detect DDTs, their approach is simplified, environmentally friendly, and sensitive. Finally, it has been possible to extend the MM/ZIF-8/IL composite concept molecules to other IL-modified MOFs and employ them to present diverse sample pretreatment procedures [139].

Konno’s study employs ZIF-8 immobilized on aramid micro-fibrils for immobilizing aramid microfibrils in order to treat wastewater. They show that mixing ZIF-8 NPs and aramid micro-fibrils results in the very slow permeation by water because of channel blockage from stacking ZIF-8 NPs. On the contrary, the high permeation rate of the nano-ZIF 8@AMF yields the acceptable permeability of water. Therefore, the handling features of ZIF-8 NPs, as adsorbents, could be promoted via immobilization on aramid micro-fibrils [140].

## 4. Conclusions

We have shown the significance of ZIFs as one of the specific subclasses of MOFs with high chemical and thermal stabilities not observed in several MOFs.

ZIFs synthesized by the traditional paths have been proposed to be valuable instruments for diverse technological uses. Previously, researchers have fabricated the as-synthesized ZIFs in deleterious and toxic organic solvents. Due to the detrimental impact of these solvents on the environment, it is possible to produce the synthetic conditions of unfavourable, harsh media and high temperature via employing green solvents, such as ethanol, which produce less pollution to the environment as compared with other solvents. Our review has provided a detailed discussion of the green strategies for synthesizing ZIFs by presenting diverse directions to devise ZIFs with lower environmental impacts and higher sustainability. Therefore, multiple variables must be investigated for assembling green MOFs, such as ambient synthesis conditions and benign solvents, such as water and bio-inspired or biomass-derived organic ligands. Moreover, our study addresses the detection methods of SEM, XRD, TGA, and so forth. Furthermore, potent attractive uses of these materials have been explored in gas separation, electrochemical performance, anti-bacterial effects, drug delivery, and so on.

It is widely accepted that the most significant concern in ZIFs’ green synthesis is the replacement of conventional solvents with safer candidates, and this scientific investigation has concentrated on the development of green ZIFs that exhibit higher space–time yield. Additionally, it is possible to modify ZIFs via making changes in the imidazole derivative, such as in the linker, the size of the pores, and substitutions of the metal nodes. Such a significant diversity implies potent design and engineering materials for other uses. Catalysis is one of the most important applications of ZIFs. These compounds are active and selective of catalysts for a wide range of reactions, from acid–base catalysis to redox catalysis. They offer new opportunities for reactions to specialized goods and products, provided that not only the nature of the active sites and the dimensions / shape of the pores can be determined, but also that the adsorption characteristics and local geometry of the active sites are also understood. In all of academic and industrial research, one of the hot topics is ZIFs’ synthesis approaches and applications. Points to consider, in addition to material design, include the challenges of synthesis approaches, such as efficiency, reaction efficiency, and related environmental impacts. Various parameters, such as water stability, cost-effectiveness, performance, biocompatibility, biodegradability, efficient, and environmentally friendly production, must be considered in the ZIF commercial market. Therefore, scientists must investigate ZIFs’ fulfilling the needs of green chemistry in order to discover their possible uses therewith. Hence, they must address available green ZIF substances for their as-yet neglected features and possible new uses.

## Figures and Tables

**Table 1:** The mixed-gas permeation features of ZIF-8 membranes at room temperature (~23 °C) with different binary gas mixtures [106].

	Permeance (10^−10^ mol m^−2^ s^−1^ Pa^−1^)						
Gas Mixture ^a^	H2	C2=	C2	C3=	C3	SF ^b^	IS ^c^
H2/H3	4360				9	545	501
H2/C3	3840			164		23	35
C2/C3			723		9	80	96
C2=/C3=		1470		165		10	13
C2=/C3		1500			9	167	190

Here, ^a^ C2, C3, C2=, and C3= represent ethane, propane, ethylene, and propylene, respectively, and ^b^ SF stands for the abbreviated separation factor. Moreover, ^c^ IS refers to the abbreviated ideal selectivity that can be computed by the permeances of a single gas.

## Data Availability

The data presented in this study are available in articles that are listed in the References.
